# Clinical outcomes of Transepithelial photorefractive keratectomy to treat low to moderate myopic astigmatism

**DOI:** 10.1186/s12886-018-0775-5

**Published:** 2018-05-09

**Authors:** Lei Xi, Chen Zhang, Yanling He

**Affiliations:** 1 Department of Ophthalmology, Peking University International Hospital, Beijing, China; 2 Tianjin Medical University Eye hospital, Tianjin Medical University Eye Institute, School of Optometry and Ophthalmology, Tianjin, China; 3 Department of Ophthalmology, Peking University People’s Hospital, Beijing, China

**Keywords:** Transepithelial photorefractive keratectomy, Myopia, Astigmatism

## Abstract

**Background:**

To evaluate the refractive and visual outcomes of Transepithelial photorefractive keratectomy (TransPRK) in the treatment of low to moderate myopic astigmatism.

**Methods:**

This retrospective study enrolled a total of 47 eyes that had undergone Transepithelial photorefractive keratectomy. Preoperative cylinder diopters ranged from − 0.75D to − 2.25D (mean − 1.11 ± 0.40D), and the sphere was between − 1.50D to − 5.75D. Visual outcomes and vector analysis of astigmatism that included error ratio (ER), correction ratio (CR), error of magnitude (EM) and error of angle (EA) were evaluated.

**Results:**

At 6 months after TransPRK, all eyes had an uncorrected distance visual acuity of 20/20 or better, no eyes lost ≥2 lines of corrected distant visual acuity (CDVA), and 93.6% had residual refractive cylinder within ±0.50D of intended correction. On vector analysis, the mean correction ratio for refractive cylinder was 1.03 ± 0.30. The mean error magnitude was − 0.04 ± 0.36. The mean error of angle was 0.44° ± 7.42°and 80.9% of eyes had axis shift within ±10°. The absolute astigmatic error of magnitude was statistically significantly correlated with the intended cylinder correction (*r* = 0.48, *P* < 0.01).

**Conclusions:**

TransPRK showed safe, effective and predictable results in the correction of low to moderate astigmatism and myopia.

## Background

Refractive errors, such as myopia and astigmatism, are the main cause of visual impairment throughout the world. A European adult population-based study found that myopia was in 35.1% of the participants and astigmatism > 0.5 cylinder diopter (D) was in 32.3% [1]. A prevalence and characteristic of corneal astigmatism (CA) in congenital cataract patients study reported that 39.25% of subjects had CA values > 2 D [2]. Another study found that 22% had CA ≥ 1.5D or higher [3].

Surgical correction of spherical and cylindrical refractive errors has led to a decrease in complications as the improvement of recent technology. Accurate correction especially astigmatism is crucial to achieve better refractive outcomes. However, treatment of astigmatism is still a challenge. Transepithelial photorefractive keratectomy (TransPRK) is popularly chosen for its flapless feature and all-in-one step procedure. Several studies have evaluated the refractive and visual outcomes after TransPRK [4–7]. As for the correction of astigmatism, refractive surgeons are concerned more on the difference between small-incision lenticule extraction (SMILE), femtosecond lenticule extraction (FLEx) and wavefront-guided LASIK [8–11]. However, very few studies have focused on the efficacy of correcting astigmatism by TransPRK, especially in the vector method [12, 13].

The aim of this study was to evaluate the results of TransPRK in the correction of low to moderate myopic astigmatism by vector method.

## Methods

### Patient population and study design

The retrospective study comprises 47 eyes of 36 patients with myopia (− 1.50 to − 5.75D) and astigmatism (− 0.75 to − 2.25D) [14] who received TransPRK between October 2016 and January 2017 at the department of ophthalmology of Peking University. The demographic data for the patients were in Table 1. All patients were provided written informed consent. The study protocol was in accordance with the Declaration of Helsinki and institutional review board.

**Table 1:** Demographic Data for the Cohort of Eyes

Parameter	Mean ± SD	Age Range
Age (y)	30.69 ± 4.85	19–38
Gender		
Male (n)	33.60 ± 3.57 (10)	26–37
Female (n)	29.58 ± 4.86 (26)	19–38

*SD* standard deviation, *SE* spherical equivalent refraction

All enrolled patients underwent a complete ophthalmic examination and had no ocular diseases except myopic astigmatism. Preoperative examinations included slit-lamp biomicroscopy, uncorrected distance visual acuity (UDVA), corrected distance visual acuity (CDVA), corneal topography (Optikon SpA, Rome, Italy), pentacam scheimpflug topography (Oculus, Wetzlar, Germany), manifest refraction, ultrasound pachymetry and dilated funduscopy examination. Patients with CDVA under 20/20, suspicion of keratoconus and thin cornea thickness were excluded.

### Surgery

The surgery was performed by a single surgeon using the SCHWIND Amaris 500E excimer laser platform (SCHWIND eye-tech-solutions GmbH, Kleinostheim, Germany). Ablations were based on aberration-free algorithms calculated using ORK-CAM software. The ablation profile targets epithelial thickness as 55 μm centrally and 65 μm peripherally according to the population model statistics [6]. Before ablation, all patients’ examinations were tested by the statistic cyclotorsion control (SCC) and dynamic cyclotorsion control (DCC) was used through the surgery. After ablation, the corneal stromal was irrigated with a cool balanced salt solution and a soft bandage contact lens was applied.

After surgery, the patients were treated with topical 0.5% levofloxacin (Cravit; Santen, Inc) eye drops four times a day for one week, 0.1% fluorometholone (Allergan, Inc) eye drops four to six times daily (tapered over 12 weeks) and preservative-free artificial tears four times daily for at least 6 months. The contact lens were removed once the epithelial closure was completed.

### Main outcome measures and vector method

The main outcomes include UDVA, CDVA, manifest refractive cylinder and sphere preoperatively and 6 months postoperatively.

**Fig. 1:**
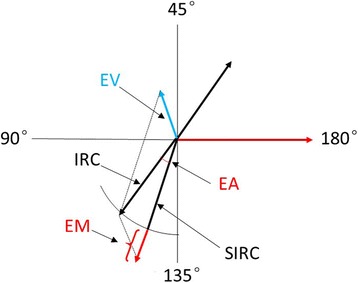
Basic astigmatic vector quantities and relationships. EA: Error of angle; EV: Error vector; EM: Error of magnitude; IRC: Intended refractive correction; SIRC: Surgically induced refractive correction

Vector analysis of corneal astigmatism is based on the definitions and formulas given by Eydelman MB [15]. The vector quantities and data used in the astigmatic analysis are shown in Fig. 1 and defined as below:

The intended refractive correction (IRC) vector: the vector difference between the preoperative astigmatic correction vector and the target postoperative cylinder vector. If the target refractive state is emmetropia, the IRC vector is equal to the preoperative astigmatic correction.

The surgical induced refractive correction (SIRC) vector: the vector difference between the preoperative and postoperative astigmatic correction vectors. SIRC is the achieved correction.

The error vector (EV): the vector difference between the IRC and SIRC (IRC-SIRC), when the refractive target is emmetropia, the EV is equal to the postoperative astigmatic correction vector.

The error of magnitude (EM): the arithmetic difference of the magnitudes between SIRC and IRC ( | IRC | - | SIRC | ).

The error of angle (EA): the angular difference between the achieved treatment and the intended treatment.

The correction ratio (CR): the ratio of the achieved correction magnitude to the required correction ( | SIRC | / | IRC | ). If the ratio = 1, it is ideal. If > 1, it means excessive application of the treatment. If < 1, it means under correction.

### Statistical analysis

Clinical data were analyzed using SPSS 20.0. Descriptive analysis with SDs for means was performed. The distribution of the data was normality, the paired Student’s *t*–test was used to analyze differences between preoperative and postoperative outcomes. Pearson correlation coefficient was used to analyze the correlation between the absolute EM and the intended cylinder correction. The relationship between the attempted refractive correction and achieved refractive correction was analyzed by linear regression analysis. A *p*-value < 0.05 was considered statistically significant.

## Result

This study included 47 eyes of 36 patients treated by TransPRK. The mean patient age was 30.69 ± 4.85 years (range: 19 to 38 years). The preoperative and postoperative visual acuity (including UDAV and CDVA) and refractive outcomes such as sphere, cylinder, and spherical equivalent were summarized in Table 2. Significant improvement was observed between preoperative and 6 months postoperatively (*p* < 0.01).

**Table 2:** Summary statistics of refractive and visual outcomes

	Preop	6 months	
	Mean ± SD (range) [median]	Mean ± SD (range) [median]	*P*-value
Sphere(D)	− 3.87 ± 1.15 (−5.75 to − 1.50) [− 4.00]	0.35 ± 0.46 (− 0.50 to 1.25) [0.25]	< 0.01
Cylinder(D)	− 1.11 ± 0.40 (− 2.25 to − 0.75) [− 1.00]	− 0.33 ± 0.25 (− 1.25 to 0.00) [− 0.25]	< 0.01
MSE(D)	−4.30 ± 1.27 (− 6.38 to − 0.88) [− 4.63]	0.18 ± 0.46 (− 0.63 to 1.13) [0.13]	< 0.01
CDVA(LogMAR)	− 0.10 ± 0.07 (− 0.20 to 0.00) [− 0.10]	− 0.14 ± 0.07 (− 0.20 to 0.00) [− 0.10]	< 0.01
UDVA(LogMAR)	0.93 ± 0.28 (0.20 to 1.50) [1.00]	− 0.10 ± 0.07 (− 0.20 to 0.00) [− 0.10]	< 0.01

*MSE* manifest spherical equivalent; *CDVA* corrected distance visual acuity; *UDVA* uncorrected distance visual acuity

### Safety and efficacy

**Fig. 2:**
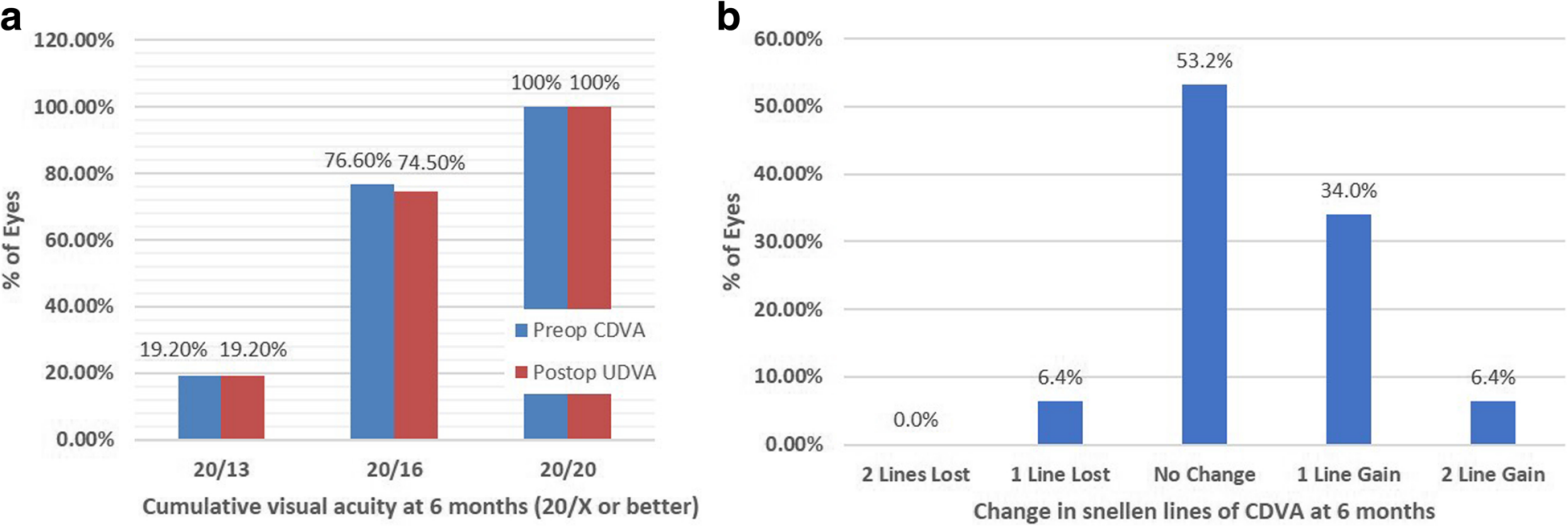
Comparison of preoperative corrected distance visual acuity (CDVA) and postoperative uncorrected distance visual acuity (UDVA) (**a**). Change in snellen lines of CDVA at 6 months postoperatively (**b**)

At 6 months postoperatively, all of the eyes achieved the UCDV of 20/20 or better visual acuity. No patient lost two or more lines of CDVA. Figure 2 shows the preoperative CDVA against postoperative UDVA and there was no significant difference between the two variables (*P* = 0.855). The changes in CDVA between preoperative and 6 months postoperative were shown in Fig. 2. 53.2% (25 eyes) of eyes unchanged; 34% of eyes (16 eyes) gain one line, 6.4% of eyes (3 eyes) gain two lines and 6.4% (3 eyes) of eyes lose one line.

### Refractive outcomes

**Fig. 3:**
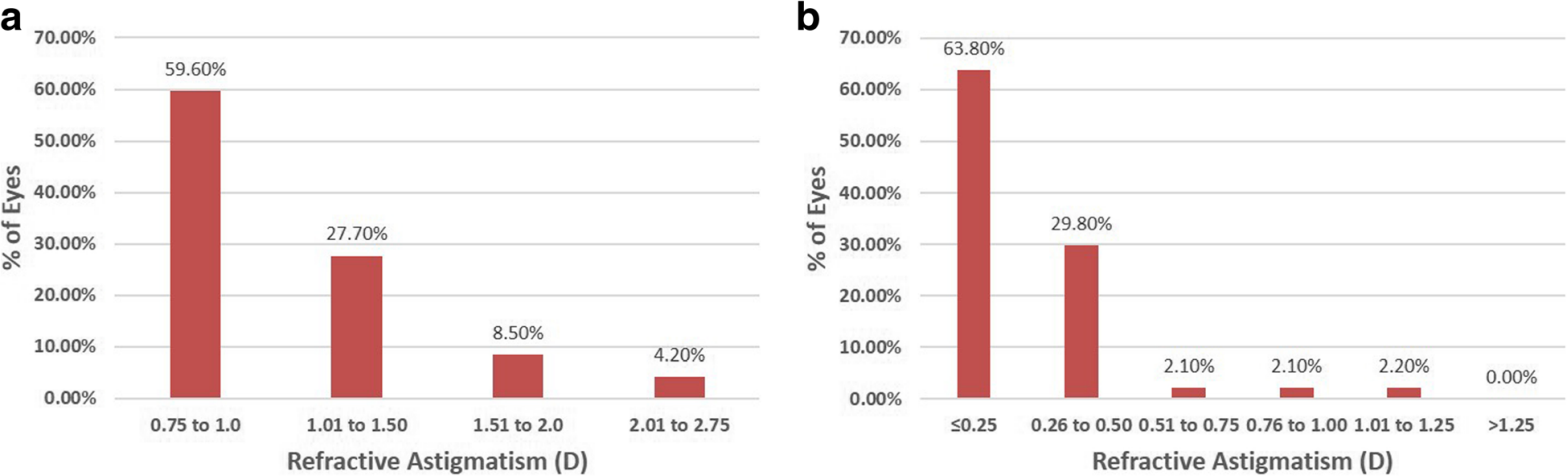
Levels of astigmatism before (**a**) and after (**b**) transepithelial photorefractive keratectomy

The preoperative and postoperative astigmatic refraction was shown in Fig. 3. 63.8% of the eyes with residual refractive cylinder ≤0.25D. Summaries of the refractive status were in Table 3. The percentage of eyes with residual refractive cylinder ≤0.50D and ≤ 1.0D was in 93.6% (44 eyes) and 97.9% (46 eyes) respectively.

**Table 3:** Summary of residual refraction

Residual magnitude	Percentage%(eyes)
0D ≤ | Sphere| ≤ 0.5D	72.3% (34)
0.5D < | Sphere| ≤ 1.0D	21.3% (10)
| Sphere|> 1.0D	6.4% (3)
0D < Cyl ≤ 0.5D	93.6% (44)
0.5D < Cyl ≤ 1D	4.3% (2)
Cyl > 1D	2.1% (1)
0D ≤ | SE | ≤ 0.5D	68.1% (32)
0.5D < | SE| ≤ 1.0D	27.7% (13)
| SE| > 1.0D	4.2% (2)

*D* diopter; *Cyl* cylinder; *SE* spherical equivalent

**Fig. 4:**
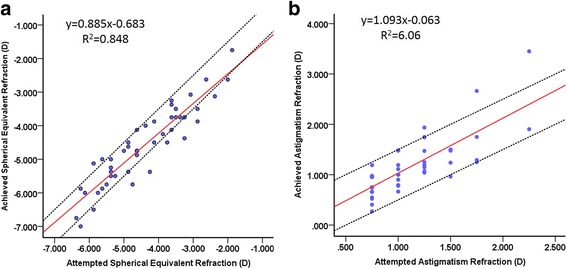
Achieved versus attempted spherical equivalent corrections 6 months postoperatively (**a**). Achieved versus attempted astigmatism corrections 6 months postoperatively (**b**). The red solid line indicates the outcome of linear regression analysis, the area between two dotted lines mean within ±0.50D

The linear regression of the scattergram was shown in Fig. 4. The achieved versus attempted spherical equivalent correction has a slop of 0.89 and the achieved versus attempted astigmatism correction has a slop of 1.09.

### Vector analysis

**Fig. 5:**
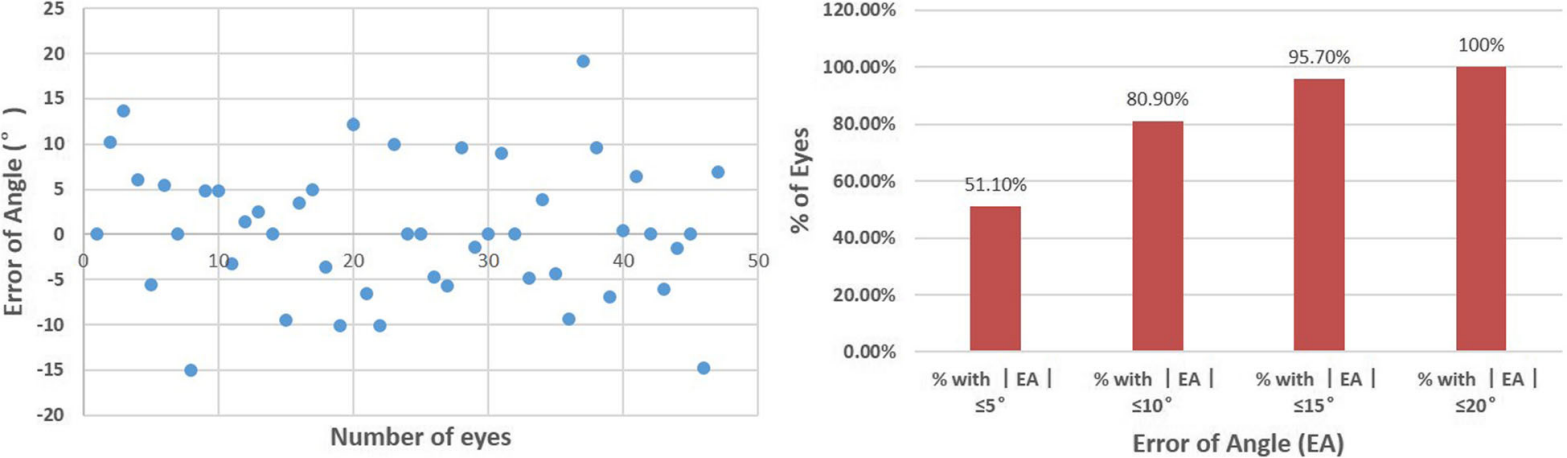
Scatter diagram of Error Angle (EA) for 47 eyes (A). Summary of the EA between the achieved treatment and the intendent treatment

Figure 5 shows the scattergram of cylinder preoperatively and the error of angle postoperatively. At 6 months, 51.1% of eyes with error of angle ≤5°and 80.9% of eyes ≤10°. No eye more than 20°.

Summarizes of all vector parameters were in Table 4. The correction ratio for cylinder was 1.03 ± 0.3. The mean error of angle (EA) was 0.44 ± 7.42°. The error of magnitude (EM) was − 0.04 ± 0.36D.

**Table 4:** Vector analysis of changes in refractive cylinder

Vector parameter	Eyes (*n* = 47) Mean (SD)	Range
IRC (D)	1.11 (0.40)	0.75–2.25
SIRC (D)	1.15 (0.57)	0.27–3.45
ER	0.30 (0.19)	0.00–0.667
CR	1.03 (0.3)	0.36–1.59
EM (D)	−0.04 (0.36)	−1.20-0.536
EA (°)	0.44 (7.42)	−15.00-19.13

*IRC* intended refractive correction; *SIRC* surgically induced refractive correction; *ER* error ratio; *CR* correction ratio; *EM* error of magnitude; *EA* error of angle

## Discussion

The evaluation of the myopic correction is significant in the clinic, especially in refractive surgery. TransPRK is recommended as one of the most advanced techniques for its flapless. Several previous studies have evaluated the safety, efficacy and predictability of TransPRK [4, 5, 13, 16]. All of the eyes had postoperative UDVA 20/20 or better. 63.8% of the eyes with residual refractive cylinder ≤0.25D, and 93.6% of the eyes≤0.50D. 80.9% of eyes with EA ≤ 5°. One study found that 70.4% of eyes were within ±0.25D and 87.0% of eyes were within ±0.50D of the attempted cylindrical correction at 3 months followed SMILE surgery [11]. In addition, another study achieved 79.1% of eyes within ±0.50D of intended correction of refractive cylinder [17]. The results of this study indicate that TransPRK in myopic eyes with low to moderate cylinder is safe, effective and predictable.

Other refractive surgeries had been analyzed in the correction of myopic astigmatism. It is difficult to compare because of the different degrees of refractive cylinder. Studies summarized in Table 5 [5, 8, 11, 12, 17–19] showed that we achieved favorable outcomes in comparison to the literature review. Adib et al. [5] reported that 97.26% of the eyes within ±0.50D 18 months later after the surgery of TransPRK for the correction of astigmatism. Similarly, Stojanovic et al. [12] achieved 79.1% of eyes within ±0.50D 12 months later after TransPRK. Zhang J et al. [17] found that 95.92% of eyes within ±0.50D followed SMILE surgery.

**Table 5:** Literature studies of myopic astigmatic correction

Author (year)	Technique	Eyes (n)	Follow-up (months)	Preoperative cylinder Mean ± SD (range)	Postoperative cylinder Mean ± SD (range)	Within 0.50D (%)
Schallhorn [17] (2015)	W-LASIK	611	3	−2.76 ± 0.81 (− 2.00 to −6.00)	−0.37 ± 0.38 (− 2.00 to 0.00)	79.10%
Stojanovic [12] (2013)	TransPRK	117	12	−0.77 ± 0.65 (−4.50 to 0.00)	/	94.00%
Adib [5] (2016)	TransPRK	146	18	−1.19 ± 0.99	−0.29 ± 0.21	97.26%
Zhang J [18] (2015)	SMILE	98	12	−0.90 ± 0.68 (− 0.25 to − 2.75)	−0.20 ± 0.27	95.92%
AI-Zeraid [19] (2016)	W-LASIK	23	6	−3.22 ± 0.59 (− 2.50 to − 4.50)	−0.72 ± 0.46	39%
Ali-MA [8] (2014)	FLEx	58	6	− 0.97 ± 0.54 (− 0.5 to − 2.75)	−0.26 ± 0.37 (− 1.00 to 0.00)	86%
Chan [11] (2015)	SMILE	54	3	−1.08 ± 0.71	−0.243 ± 0.316	87%
Current study	TransPRK	47	6	−1.11 ± 0.40 (−0.75 to − 2.25)	− 0.33 ± 0.25 (− 1.25 to 0.00)	93.6%

*W-LASIK* wavefront-guided Laser in situ keratomileusis; *TransPRK* transepithelial photorefractive keratectomy; *SMIL* small-incision lenticule extraction; *FLEx* femtosecond lenticule extraction

Previous studies have attempted to evaluate the refractive outcomes by the spherical equivalent (SE). It is less precise because astigmatism includes both magnitude and axis direction. For this reason, vector analysis could describe the astigmatism correctly. Very few refractive surgery results such as small-incision lenticule extraction (SMILE), femtosecond lenticule extraction (FLEx) and LASIK had been analyzed in the vector method [7, 9, 11, 18, 20]. The aim of this study is to investigate the correction of low to moderate myopic astigmatism following TransPRK in vector method, which has not been reported until now.

The vector analysis of refractive cylinder showed slight overcorrection with the correction ratio (CR, ratio of the achieved correction magnitude to the required correction) of 1.03 at 6 months postoperatively. + 9% overcorrection was observed in the linear regression of the attempted astigmatism refraction. The error of angle (EA) was anticlockwise and minimal (0.44°). Compare to other studies that use vector analysis of cylinder correction, Schallhorn et al. [7] found slight under correction of the refractive cylinder and a mean EA of − 0.45°following wavefront-guided LASIK in the treatment of moderate-to-high astigmatism. Katz et al. [21] achieved a similar CA of 1.06 to our study, and the EA of 3.60°in eyes with preoperative refractive cylinder greater than 3.00D. Chan et al. [11] found EA of − 0.85°and 2.09°in SMILE and femtosecond-assisted LASIK respectively in the correction of low to moderate myopic astigmatism. In this study, the error ratio of cylinder correction is 0.30, which is slightly higher than 0.13 reported by Schallhorn et al. [7]. These results indicate that TransPRK could achieve comparable results in the correction of cylinder by comparing with other surgeries. Statistic cyclotorsion control (SCC) and dynamic cyclotorsion control (DCC) may play an important role in the correction of cylinder.

The alignment is important for the refractive cylinder correction. Misalignment could result in both higher order aberrations (HOAs) and corrective performance [18, 22]. In our study, 80.9% of eyes within the error of angle (EA) ≤10°, and 95.7% within ≤15°. One study found 68.4% of eyes had EA ≤10°in a group of patients with preoperative refractive cylinder of − 0.90 ± 0.68D followed SMILE surgery [18]. Another study found 98.4% with EA within 10°after wavefront-guided LASIK [7]. Although eye-tracking was used for every patient, the astigmatism axis could be misaligned by many factors. The cyclotorsional movements of the eyes from upright to supine position, the shift of the pupil center and the large angle kappa occurred. In addition, the epithelial thickness (55 μm centrally and 65 μm periphery) estimated by the statistic of common people may lead to deep or shallow ablation of the corneal tissue. So, precise positioning center and epithelial mapping for every patient would be of great help for the precise correction of refractive cylinder.

In conclusion, the outcomes of this study suggest that TransPRK surgery is safe, effective and predictive in the treatment of low to moderate astigmatism. Based on the findings of our study, the absolute astigmatic error of magnitude was significantly correlated with the intended cylinder correction. Further investigation should be conducted to assess the treatment for correcting higher cylinder.

## Conclusions

TransPRK surgery is safe, effective and predictable in the correction of low to moderate astigmatism.

## Abbreviations

CA: Corneal astigmatism
CDVA: Corrected distance visual acuity
CR: Correction ratio
DCC: Dynamic cyclotorsion control
EA: Error of angle
EM: Error of magnitude
ER: Error ratio
EV: Error vector
HOAs: Higher order wavefront aberrations
IRC: Intended refractive correction
SCC: Statistic cyclotorsion control
SE: Spherical equivalent
TransPRK: Transepithelial photorefractive keratectomy
UDVA: Uncorrected distance visual acuity
